# Peritoneal inclusion cyst causing intestinal obstruction in a 9-year-old girl: A rare pediatric case

**DOI:** 10.1016/j.radcr.2025.10.044

**Published:** 2025-11-18

**Authors:** Tasneem Jaabou, Husam Tarakhan, Mohamed Bashagha, Mohammad Alzu’bi, Muataz Kashbour, Wasan Hayajneh

**Affiliations:** a Department of General and Pediatric Surgery, Ministry of Health, Amman, Jordan; b Faculty of Medicine, The Hashemite University, Zarqa, Jordan; c Radiology Department, Faculty of Medicine, Misurata University, Misrata, Libya; d Diagnostic Radiology Department, National Cancer Institute-Misrata, Misrata, Libya; e Department of Pathology, Ministry of Health, Amman, Jordan.

**Keywords:** Case report, Peritoneal inclusion cyst, Intestinal obstruction, Pediatric

## Abstract

Peritoneal inclusion cyst (PIC) is a rare benign condition with low-risk malignant transformation that typically occurs in adult women with a history of pelvic inflammatory conditions, endometriosis, or prior abdominal surgery. Cases in pediatrics are extremely rare. We present a 9-year-old female with acute intestinal obstruction due to PIC, characterized by a 1-week history of severe colicky abdominal pain localized to the suprapubic area and radiating to both flanks with abdominal distension, and 4 days of vomiting. A computed tomography (CT) scan shows a large multilocular cystic mass occupying the entire abdominal cavity and compressing adjacent organs. An emergent laparotomy was performed, resulting in successful cyst resection. Histopathology confirms the diagnosis of PIC. The patient recovered well postoperatively, and recurrent follow-up over the next year revealed that she remained asymptomatic, with normal physical examination and vital signs. This case emphasizes the importance of considering PICs in the differential diagnosis for pediatric patients presented with intestinal obstruction, even in the absence of typical risk factors. It also highlights the value of early diagnosis and timely surgical intervention for symptomatic cases.

## Introduction

Peritoneal inclusion cysts (PICs) are benign, reactive cystic formations within the peritoneal cavity. They develop due to impaired peritoneal fluid absorption, typically secondary to developmental anomalies, trauma, inflammation, postsurgical adhesions, or endometriosis [1]. As the peritoneum's fluid absorption capacity diminishes, reactive adhesions and scar tissue form cysts or cavities that trap the serous fluid [1]. Since first being described by Mennemeyer in 1979, PICs have been recognized more frequently reported in adult women; however, their occurrence in pediatric patients remains rare and often underdiagnosed [2,3].

The clinical presentation of PICs is diverse, ranging from asymptomatic cases identified incidentally on imaging studies to symptomatic cases presenting with abdominal pain, distention, nausea, and vomiting [3,4]. Diagnosis typically relies on imaging modalities such as ultrasound and MRI. Surgical intervention is generally reserved for symptomatic cases or when there is concern for malignancy [3,5].

This report presents a rare case of PIC in a 9-year-old female, which highlights the importance of increasing clinician awareness regarding the diagnosis and management of PICs in pediatric patients.

## Case presentation

A 9-year-old girl presented to the emergency department with a 1-week history of severe colicky abdominal pain and distension, along with 4 days of vomiting. She had no history of previous surgery and had not yet attained menarche. Initially, vomiting occurred after any oral intake, containing undigested food and gastric juice; it later became bloody. The abdominal pain was colicky and intermittent, localized to the suprapubic region, and radiating to both flanks.

During physical examination, the patient appeared ill, lethargic and in pain; her vital signs were as follows: heart rate was 100 bpm, blood pressure was 100/70 mmHg, respiratory rate was 20 breaths per minute, and temperature was 37°C.The abdominal examination revealed asymmetry, enlargement, and a palpable midline suprapubic mass extending from the suprapubic region to the epigastric area. Digital rectum examination revealed an empty rectum.

**Fig. 1:**
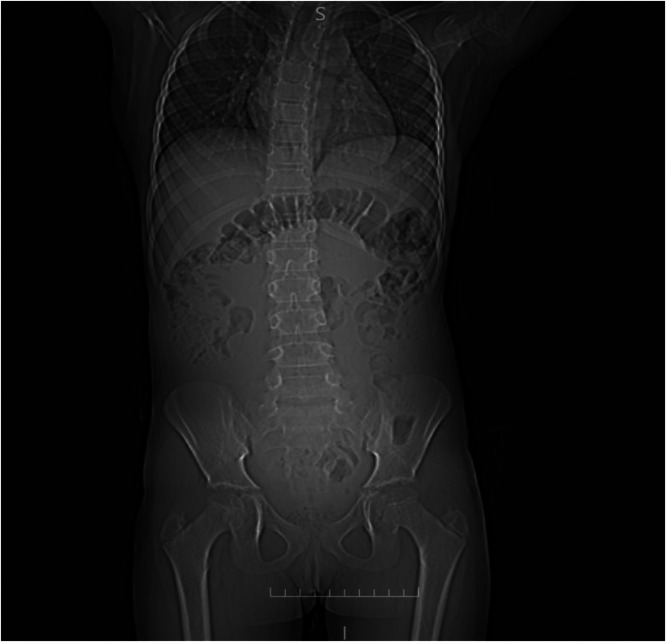
Supine abdominal radiograph demonstrating a large soft-tissue opacity with upward displacement of gas-filled transverse colon and nondilated small-bowel loops.

**Fig. 2:**
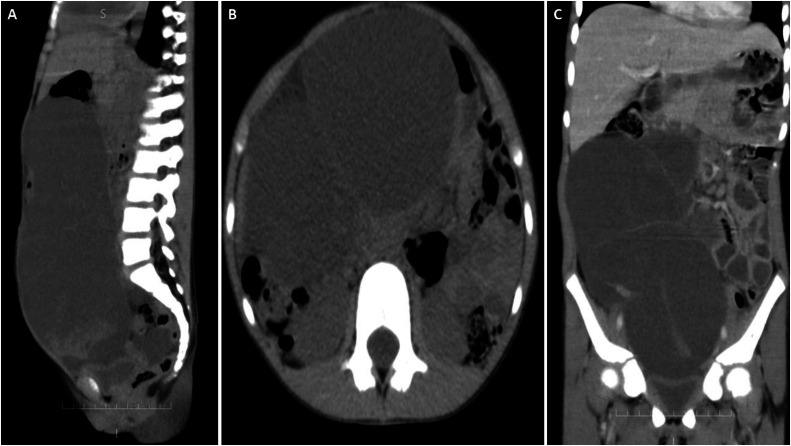
Contrast-enhanced CT of the abdomen and pelvis (portal venous phase) demonstrating a large, multiloculated, cystic lesion with internal septations occupying the abdominal cavity and compressing adjacent organs: (A) Sagittal cross-section. (B) Axial cross-section. (C) Coronal cross-section.

Plain abdominal radiography demonstrated a large soft-tissue opacity with upward displacement of the gas-filled transverse colon and nondilated small-bowel loops. No air-fluid levels were identified (Fig. 1). Ultrasound imaging revealed a heterogeneous mass extending from the suprapubic area to the epigastric area with minimal free fluid in the pelvis. Subsequent computed tomography (CT) scan with intravenous contrast revealed a huge multilocular cystic lesion with internal septations occupying the entire abdominal cavity and compressing adjacent organs with unclear origin. The lesion displaced, rather than infiltrated, adjacent bowel loops and showed no appreciable wall or septal enhancement on contrast-enhanced CT (Fig. 2). No abnormalities were noted in the liver, pancreas, spleen, kidneys, or adrenal glands.

**Fig. 3:**
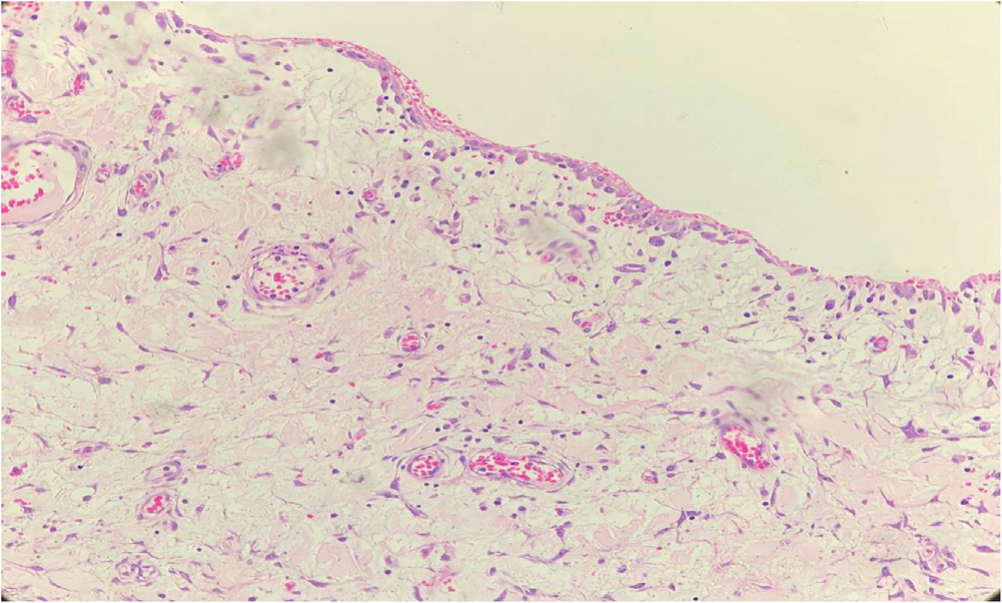
Higher power view (200X): Cyst wall composed of loose myxoid and collagenous stroma lined by single layer of bland mesothelial cells seen on the top.

Decompression via a nasogastric tube was initiated, and the patient underwent emergent laparotomy. Intraoperatively, a large cystic mass was identified and completely resected. Gross examination revealed a cyst that measures 8.0 × 4.5 cm attached to the outer wall of a jejunal segment, without vascular compromise. Microscopic examination of the resected specimen revealed that the cyst wall is composed of loose myxoid collagenous tissue lined by a single layer of bland mesothelial cells. Also, the resected specimen contains a segment of small bowel with an attached cyst wall involving the outer serosal and subserosal layers. No evidence of granuloma, dysplasia, or malignancy. These findings were consistent with a diagnosis of PICs (Fig. 3).

For further mass evaluation, serum tumor markers were evaluated including alpha-fetoprotein (AFP), beta-human chorionic gonadotropin (B-HCG), cancer antigen 125 (CA 125), cancer antigen 19-9 (CA 19-9), and cancer antigen 15-3 (CA 15-3), to help differentiate potential etiologies. AFP and B-HCG were tested to rule out germ cell tumors, CA-125 to evaluate the likelihood of ovarian malignancy, CA 19-9 to exclude gastrointestinal or other abdominal malignancies, and CA 15-3 to assess the potential for ovarian or other abdominal tumors. Among these markers, only CA-125 was elevated, with a level of 78.185 U/mL (normal level < 35 U/mL). However, CA-125 is a nonspecific marker, and may be elevated in both benign and malignant conditions, as well as in various inflammatory and physiological processes.

The patient had an uneventful postoperative recovery. During follow-up over 1 year, she remained asymptomatic with normal physical examinations and stable vital signs.

## Discussion

This report presented the first documented pediatric case of PICs causing intestinal obstruction in the absence of prior abdominal surgery. Notably, this is only the second reported case of an inclusion cyst presenting with intestinal obstruction in the literature. The previously reported case involved an adult female with a history of laparotomy following trauma, which are well established risk factors for the development of PICs [6,7].

Although PICs are more prevalent in adult women, particularly those within the reproductive age range (mean age approximately 34 years), and are associated with a history of prior abdominal surgery or chronic pelvic conditions such as endometriosis or pelvic inflammatory disease, this case emphasizes the importance of considering PICs in the differential diagnosis of pediatric patients presenting with intestinal obstruction, even in the absence of these typical risk factors [4,8,9]. The pathogenesis of PICs in pediatric patients is still not well understood. Unlike adults, children rarely have risk factors such as surgery, endometriosis, or pelvic inflammatory disease. It is hypothesized that congenital abnormalities of the peritoneum, localized developmental anomalies, or unrecognized low-grade inflammation may play a role in the formation of cysts in children.

Diagnosis of PIC can be challenging due to nonspecific symptoms. Vague pelvic pain or diffuse abdominal pain being the most common presentation, with other reported symptoms were abdominal distension, constipation, and recurrent urinary symptoms [7]. The clinical presentation of PICs often depends on their size and location, with larger cysts potentially compressing surrounding structures and leading to acute symptoms such as intestinal obstruction, as observed in our case [4,10]. Given the prevalence of cystic lesions in the postpubertal female which is the most common age of PIC, the list of differential diagnoses is extensive when such lesions are detected, including ovarian cancer [11]. The nonspecific clinical features of PICs can mimic various more prevalent abdominal disorders in children, such as appendicitis, gastrointestinal duplication cysts, ovarian torsion, or even malignancy. Although a definitive diagnosis of PIC requires histopathology, earlier consideration of PIC in the differential diagnosis can help guide intraoperative decision-making towards conservative surgical goals, such as complete cyst excision and adhesiolysis with preservation of bowel and ovarian tissue. In pediatric patients with abdominal pain, distension, and vomiting, timely imaging and surgical exploration remain the standard of care regardless of preoperative suspicion.

Preoperative assessment of pelvic masses often involves the evaluation of malignancy risk using parameters such as tumor markers, ultrasound features, and color Doppler ultrasonography [12]. Ultrasound, either transabdominal and or transvaginal, is the initial imaging modality. PICs typically appear as anechoic, multiseptated cystic structures with absent internal vascularity on color Doppler ultrasound [13].

Cross sectional imaging including computer tomography and magnetic resonance imaging is required for further evaluation. These modalities show intraabdominal, primarily pelvic, single or multilocular cystic lesions [13]. This can lead to a broad differential list in the female pelvis including both benign and malignant conditions. MRI is particularly helpful in determining the origin of the lesion and if there is any solid [13,14]. Differential diagnoses during workup include mucinous cystic neoplasm, lymphangiomas, enteric duplication cysts, adenomatoid tumors, and both benign and malignant ovarian tumors [14].

Management of PICs remains controversial ranges from observation to complete surgical resection. While surveillance or aspiration may be considered for small, asymptomatic cysts in some cases, most advocate surgery as the primary treatment. Given the high recurrence rates (45-50%) and potential for malignant transformation, optimal surgical management often includes cytoreduction to remove gross disease, sometimes complemented by hyperthermic intraperitoneal chemotherapy to address microscopic residual disease [14,15]. However, aggressive treatment may not always be necessary or appropriate, especially in reproductive-age women where fertility concerns are paramount [16]. Finally, planning a follow-up strategy is crucial, particularly given the documented risk of recurrence. Given its benign nature and the desire to minimize radiation in a pediatric population, a prudent strategy involves regular clinical assessment and serial follow-up with ultrasound to monitor for any signs of recurrence.

## Conclusion

This case report describes a rare occurrence of PICs causing intestinal obstruction in a pediatric patient with no history of abdominal surgery or pelvic pathology. Although PICs are typically seen in adult women with identifiable risk factors, this case highlights the importance of considering PICs within the differential diagnosis of pediatric patients presenting with obstruction symptoms. Successful surgical resection of the cyst underscores the importance of early diagnosis and appropriate surgical intervention for symptomatic PICs. Further research is needed to better understand the incidence, the pathophysiology and clinical presentation of PICs in pediatric populations, allowing for improved diagnostic accuracy and the development of optimized management strategies.

## Authorship

All authors attest that they meet the current ICMJE criteria for Authorship.

## Patient consent

We confirm that written informed consent was obtained from the patient’s parents in their native language for the publication of this case report. The parents were fully informed about the details to be published and understand the implications of publication.
